# A pilot study of cutaneous oxygenation and perfusion in systemic sclerosis–related digital calcinosis

**DOI:** 10.1093/rheumatology/keaa131

**Published:** 2020-04-07

**Authors:** Joanne B Manning, Elizabeth Marjanovic, Tonia L Moore, Graham Dinsdale, Sarah Wilkinson, Mark R Dickinson, Ariane L Herrick, Andrea K Murray

**Affiliations:** k1 Division of Musculoskeletal and Dermatological Sciences, Salford Royal NHS Foundation Trust, Manchester Academic Health Science Centre; k2 Photon Science Institute; k3 Department of Physics and Astronomy, University of Manchester, Manchester, UK

**Rheumatology key message**

- Decreased perfusion at SSc-related calcinosis sites supports the concept that ischaemia drives calcinosis development.

Sir, Up to a quarter of patients with SSc will develop s.c. calcinotic deposits, which are often painful and can perforate the skin, causing ulceration [1–3]. The underlying aetiology is unknown and there is currently no effective treatment. Our previous study assessing plain radiographs for the presence of acro-osteolysis and calcinosis suggested a link between severe digital ischaemia (assessed by previous in-patient intravenous prostanoid therapy, surgical debridement or amputation of the digits), acro-osteolysis and calcinosis [4]. Others have also suggested that ischaemia may contribute to calcinosis development [2, 5]. The aim of this study was to elucidate the pathophysiology of SSc-related calcinosis using non-invasive imaging modalities that allow the measurement of oxygenation and blood flow (perfusion) around sites of calcinosis. Specifically, the study tested the hypotheses that hypoxia and ischaemia ‘drive’ the development of calcium deposition; the severity of oxygenation and perfusion changes are related to the size and depth of the calcinosis.

Twenty-one patients with SSc-related calcinosis of the hands participated in the study. All were female, with a median age of 63 years [interquartile range (IQR) 55–70], disease duration since onset of first non-RP feature 14 years (IQR 9–23), RP duration 23 years (IQR 12–36), 18 with lcSSc, 3 with dcSSc and 16 ACA positive. Images were obtained at the site of the calcinosis and at an adjacent site of unaffected skin (Fig. 1). Measurements of oxygenation were extracted from images taken by a bespoke multispectral imager and broadband light source, taking several images over multiple wavelengths in order to calculate oxygenation based on deoxyhaemoglobin and oxyhaemoglobin absorption spectra (as described previously [6]). Perfusion was measured from images taken by three imaging techniques: thermography, a pseudo-measure of perfusion (FLIR ONE, FLIR Systems, Täby, Sweden) that images the vasculature of the skin and upper layers ofthe underlying superficial muscle; laser Doppler imaging (MOORLDI2, Moor Instruments, Devon, UK), whichmeasures superficial and deeper cutaneous microvascular levels; and laser speckle contrast imaging (MOORFLPI2, Moor Instruments), which images the upper levels of the cutaneous microcirculation. Lesion area and depth were assessed by high-resolution US images in order to determine the size and depth of the lesion (US imaging of calcinosis has been previously reported as a possible alternative to plain radiography for demonstrating/measuring calcinosis [7, 8]).

**Fig. 1:** Photograph/perfusion imaging of digital calcinosis alongside boxplot of perfusion at the site of calcinosis and an adjacent unaffected site 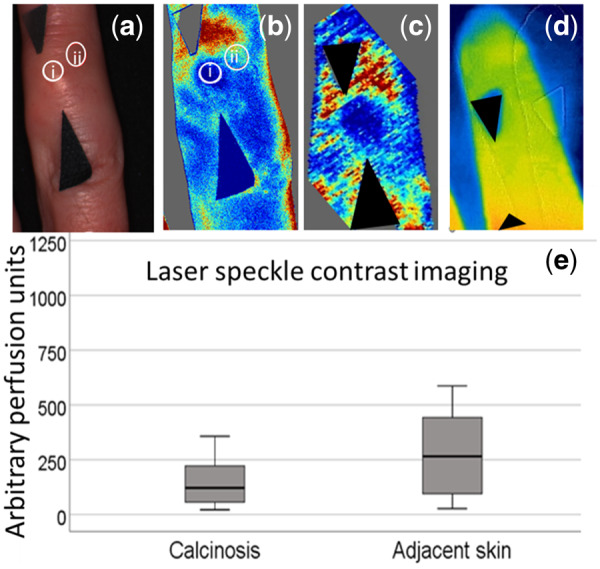
(**a**) Photograph of a finger with s.c. calcinosis. Circles indicate regions of interest at the site of calcinosis and an adjacent site. (**b**) Laser speckle with corresponding regions of interest to (**a**) marked. (**c**) Laser Doppler image. (**d**) Thermal image. For (**b–d**), arrows indicate the site of calcinosis and blue is relatively low perfusion/temperature and red high perfusion/temperature. (**e**) Box plot of data from calcinosis site and adjacent site measured by laser speckle imaging. Central line is the median value, box outline is IQR and whiskers are maximum and minimum excluding outliers.

Wilcoxon signed rank tests were performed to examine whether there was a significant difference between measurements obtained at the site of calcinosis and the adjacent skin site. Spearman’s ρ correlations were performed to examine the relationships between measurements. The study complied with the Declaration of Helsinki and was approved by the North-West Research Ethics Committee 6. All participants gave written consent.

Twenty-one lesions were imaged, one calcinotic lesion from each patient (example shown in Fig. 1). One patient did not undergo laser speckle contrast imaging due to technical reasons. Two patients did not have US images due to having open wounds. Due to the availability ofequipment, only 14 lesions were imaged for oxygenation.

There was no difference in the oxygenation at the site of calcinosis *vs* the adjacent site (*n* = 14): median 0.15 [inter-quartile range (IQR) 0.07–0.22] *vs* 0.16 (0.00–0.21) arbitrary units, respectively, *P* = 0.38. Skin temperature as imaged by thermal camera was similar between all calcinosis and adjacent sites (*n* = 21): 31.4°C (IQR 28.4–35.6) *vs* 32.8 (28.4–35.7), *P* = 0.052. Perfusion as measured by laser Doppler imaging was lower in 13 of 21 calcinosis sites as compared with the adjacent site, but grouped comparison of perfusion was not significantly decreased [349.0 (IQR 111.05–757.7) *vs* 390.9 (156.3–653.0), *P* = 0.64]. However, perfusion as measured by laser speckle contrast imaging was reduced at 19 of 20 calcinosis sites as compared with adjacent sites [121.6 (IQR 55.7–226.4) *vs* 265.4 (89.6–446.9) arbitrary perfusion units, *P* < 0.01] (Fig. 1 shows images, arrows indicating calcinosis, blue representing relatively low perfusion/temperature). The median depth of the calcinoses was 1.5 mm (IQR 1.11–2.07) and the median lesion cross-sectional area was 3.06 mm^2^ (IQR 2.33–4.62). There were no relationships (Spearman rank correlations were non-significant) between the size and depth of the calcinosis and oxygenation or perfusion.

In conclusion, laser speckle contrast imaging, which images superficial skin layers, demonstrated significant differences in perfusion between calcinotic and adjacent area perfusion and so our findings provide further support for an ischaemic contribution to calcinosis formation. Laser Doppler imaging and thermography that imaged deeper layers of the skin and s.c. tissue showed a trend towards decreased skin perfusion, but indicated that deeper layers of the skin are potentially less affected (than superficial layers) by ischaemia at calcinotic sites. That perfusion was decreased in the area of the calcinosis *vs* the adjacent skin may be due to pressure effects on the skin leading to ischaemia (calcinosis typically occurs at pressure points), or it may be that calcinosis develops in areas that are underperfused for other reasons.
